# Development of an Ankle Exoskeleton: Design, Modeling, and Testing

**DOI:** 10.3390/s25072020

**Published:** 2025-03-24

**Authors:** Gani Sergazin, Assylbek Ozhiken, Nursultan Zhetenbayev, Kassymbek Ozhikenov, Gulzhamal Tursunbayeva, Yerkebulan Nurgizat, Arman Uzbekbayev, Abu-Alim Ayazbay

**Affiliations:** 1 Department Global Education and Training (GET), University of Illinois at Urbana-Champaign, Champaign, IL 61820, USA; sergazin@illinois.edu; 2 Institute of Mechanics and Engineering named after Academician U.A. Dzholdasbekova, Almaty 050013, Kazakhstan; 3 Department of Robotics and Technical Tools of Automation, Satbayev University, Almaty 050013, Kazakhstan; n.zhetenbaev@aues.kz (N.Z.); ozhikenovk@gmail.com (K.O.); y.nurgizat@aues.kz (Y.N.); niipntkz@gmail.com (A.U.); work_abu@hotmail.com (A.-A.A.); 4 Department of Information Security, Eurasian National University, Astana 10000, Kazakhstan; t.gulzhamal@outlook.com

**Keywords:** exoskeleton, rehabilitation, ankle joint, kinematics, biomechanics

## Abstract

This research presents the results of conceptual design and modeling of an exoskeleton. It is intended for ankle joint rehabilitation in patients with musculoskeletal disorders. The exoskeleton design includes three screw actuators that smoothly control motion in the planes of dorsal and plantar flexion, inversion, and eversion. The results of the virtual tests performed on the exoskeleton device demonstrated a high degree of adaptability to varying loads and different phases of motion. Controlled torque fluctuations and linear motion provide the necessary support during different phases of rehabilitation, which has a positive impact on the patient’s recovery rate. The advantages of the design include material availability, ease of use, and flexibility in customization, making it an attractive option for use in both clinical and home settings. The study emphasizes the importance of developing affordable and accurate rehabilitation devices that can adapt to individual patient needs.

## 1. Introduction

Ensuring healthy lifestyles and well-being for all age groups is one of the main strategies for achieving the Sustainable Development Goals. In this case, the rehabilitation process becomes an important element in restoring health and improving the quality of life of people by providing universal access to health care [1]. According to World Health Organization (WHO) data, about 2.4 billion people worldwide today have diseases that require rehabilitation interventions. The WHO predicts that this need will increase due to changes in health care and demographics worldwide [2]. Given the increasing number of patients with musculoskeletal disorders, especially in the elderly [3], a personalized and precise approach to the rehabilitation process is an important challenge.

In the musculoskeletal system, the ankle joint plays a crucial role in maintaining mobility and stability [4]. It is characterized by a complex structure that is subjected to high loads and flexible movements daily. Any anomalies resulting from ankle joint trauma, including degenerative diseases such as osteoarthritis or stroke sequelae, significantly limit the quality of life of patients [5]. Traditional ankle physiotherapy methods are often not precise enough, and existing solutions in the form of rehabilitation devices face adaptability problems, high cost, and high energy consumption. These limitations actualize the development of devices that combine accuracy, affordability, and biomechanical compatibility.

Consequently, researchers around the world are developing different types of rehabilitation devices using a wide range of methods and solutions. Today, among their varieties, robotic exoskeletons have found widespread use and are becoming a very valuable working tool for physiotherapists and orthopedists [6].

The most significant advantage of exoskeletons is the possibility of performing simple, repetitive, and intensive movements in the process of rehabilitation therapy. It is also noted that they can be performed without the physical presence of a specialist. This is confirmed by studies [7,8,9,10], which demonstrate positive results of rehabilitation processes. In this case, the authors developed several varieties of exoskeletons and conducted experiments with the application of various rehabilitation programs and training in real conditions with patients with disorders in the musculoskeletal system. Experimental study of the devices has shown promising results in rehabilitation processes, but finding a suitable configuration to adjust and adapt the device to the needs of each individual case remains a challenge.

Many studies [11,12,13,14,15] have focused on the development and testing of exoskeletons for ankle rehabilitation. Some interesting conclusions were drawn from the results obtained. The developed prototypes effectively assisted ankle joint movement and rehabilitation through a novel design, showing reasonably good performance and biomechanical compatibility. The difference between them may be that the authors applied different approaches and dynamic models including algorithms to control the exoskeleton. In some studies [16], the authors have made efforts to develop a soft exoskeleton that can help patients with lower limb impairments. In this case, the presence of a flexible actuator unit with a Bowden cable significantly increases the efficiency of force transmission during plantar flexion of the ankle joint. The developed device provides natural mobility, which is confirmed by the obtained results of physiological, metabolic, and muscle activation tests. However, the system has a limited loading capacity. Some authors have contributed to the development of exoskeleton control systems. Among them, we can emphasize a study [17] where an innovative approach is studied by processing data using neural networks. The tests performed demonstrated high performance, which further facilitates the reliable estimation of gait kinematic parameters and reduces the dependence on a large set of sensors. However, the neural network must initially be trained using computational data such as leg angles, angular velocities, etc. Another interesting study [18] in this area was dedicated to the development of a lower limb exoskeleton with adaptive iterative control for motion trajectory tracking. The authors in this paper propose an algorithm based on an online linear quadratic regulator with iterative learning (OILLQR). The obtained results confirm the good tracking performance, but the online learning for each trajectory point is a time-consuming process.

Currently, many studies focus on open questions and propose solutions for new ankle rehabilitation technologies, such as being equipped with a safe automatic stretching machine that improves ankle flexibility, a precise monitoring device, or pressure sensors to track movements [19,20,21,22,23,24,25]. Overall, rapid advances in ankle rehabilitation technologies are emphasized, including innovative devices, low-cost devices, and accurate monitoring tools that can improve the effectiveness of rehabilitation. To date, current research into rehabilitation exoskeletons shows great progress, but key gaps remain unresolved. For example, many tether-driven devices, such as exosuits, provide natural mobility but have limited payload capacity. Established AI-enabled hybrid systems, while adaptive, require significant computational resources and are difficult to clinically customize. In addition, many solutions focus on a single plane of motion, ignoring the complex biomechanics of the ankle joint. These shortcomings highlight the need to develop an exoskeleton that combines multi-axis mobility, energy efficiency, and customization.

The development of new rehabilitation devices may be the current requirement of rapidly changing technologies in the world. All the studies discussed above are evidence of the current trend. Many advanced control systems, mechanisms, sensors, etc. have been developed. All the studies conducted can be a good background for the development of a new rehabilitation robotic exoskeleton, which is presented in this paper.

This research focuses on the development of an innovative exoskeleton designed for ankle joint rehabilitation. The exoskeleton has three screw-based actuators that provide a self-locking transmission. This eliminates the need for the motors to run continuously to fix the joint. The presence of triplanar control gives the ability to reproduce natural movements such as rear flexion/cambulatory flexion and inversion/extension. The efficient design using available materials, PLA plastic, and an Arduino controller significantly reduces the weight of the device and makes it suitable for use in a wide range of rehabilitation settings.

The main objective of the study is to develop and validate an innovative exoskeleton. The work evaluates the ability to adapt the exoskeleton to the individual biomechanical parameters of patients with ankle joint disorders and injuries. The results of the study include kinematic modeling using SolidWorks Simulation (SolidWorks Simulation Professional 2023). A biomechanical analysis of the ankle joint range of motion was also performed. Virtual tests for stability and control accuracy of the exoskeleton were performed. The article is structured in the following sections: an introduction together with a review of existing research, a description of the design methodology, a description of the modeling results and virtual tests with a discussion of clinical perspectives, and a section of key findings and future research directions.

## 2. Materials and Methods

This study proposes a novel robotic exoskeleton. It can be applied as an ankle joint rehabilitation device. The exoskeleton is designed based on the biomechanical features of the ankle joint to improve their bionic characteristics. Thus, the physiological structures of human lower limb joints were analyzed, and the kinematic chain of the human lower limb was established. In order to calculate the necessary parameters of the 3D model of the prototype, a kinematic circuit was developed, which makes it possible to carry out mathematical modeling and determine the degrees of freedom of the device.

### 2.1. Study of Biomechanical Characteristics of Ankle Joint

**Figure 1:**
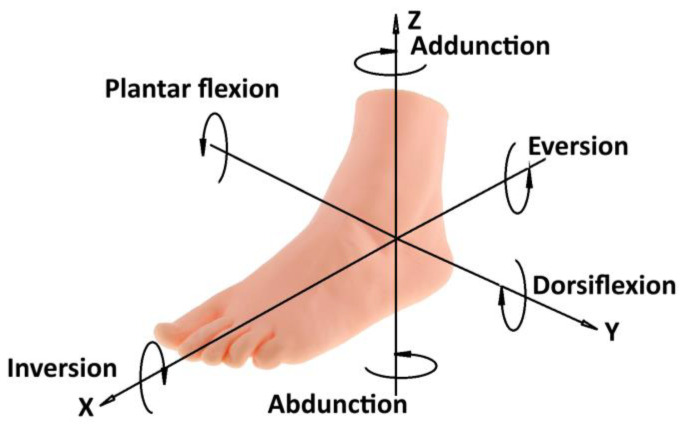
Foot mobility diagram.

As can be known from anatomy, the mobility of the foot is defined in 3 motions, which are shown as a foot mobility diagram in Figure 1. It shows the basic movements of the foot in relation to the three axes of the X, Y, and Z coordinates.

Plantar flexion is a downward movement of the foot along the X-axis toward the sole. It involves the contraction of the posterior tibial muscles, which causes the toes and soles to lower. Dorsiflexion occurs along the Y-axis and is an upward movement, bringing the foot closer to the tibia. This movement is accomplished by the contraction of the muscles in the anterior tibial group, lifting the toes and the back of the foot. Inversion is an inward rotation of the foot around the X-axis, where the sole turns medially. This movement is performed by the muscles on the medial side of the foot and tibia. Eversion is an outward rotation of the foot around the Y-axis, where the sole turns laterally, accomplished by the muscles on the lateral side of the foot and tibia. Adduction is a movement around the Z-axis in which the foot moves inward, approaching the midline of the body. This type of movement helps stabilize the foot under various loads and is supported by the medial muscles of the foot. Abduction also occurs around the Z-axis and is an outward movement of the foot away from the midline of the body. Abduction is important for balance and is accomplished by the contraction of the lateral muscles of the foot.

The biomechanical features of the ankle joint consist of a set of interrelated characteristics that determine its function and performance. This joint comprises three bony components, the ankle (calcaneus), the bones of the foot (tarsals), and the heel (phalanges), as well as a ligamentous apparatus and muscles, which ensure its stability and mobility.

The biomechanical features of the ankle joint consist of a set of interrelated three bony components, the ankle joint (calcaneus), the bones of the foot (tarsal) and heel (phalanx), and the ligamentous apparatus and muscles that provide its stability and mobility. These components form a complex mechanical system that includes rotational axes, contact surfaces, and the ligaments and muscles that control movement, such as dorsiflexion, plantar flexion, and internal and external rotation. The functional role of the ankle joint is to support body weight, transfer loads to other joints, and participate in various types of movement. The mechanical behavior of the ankle joint is characterized by parameters such as flexibility, elasticity, stability, and the ability to adapt to different types of loads. Its dynamic behavior is characterized by properties such as mass movements, energy efficiency, and temporal characteristics of movement. Physiological processes affecting joint function include muscle contraction and relaxation, physiological stiffness, and synovial fluid release. These processes play a key role in maintaining normal joint function [26].

Understanding these biomechanical features is crucial for the diagnosis and treatment of ankle joint diseases and injuries, as well as for the development of exoskeletons that create support and correction systems. Analyzing these characteristics allows for a better understanding of ankle joint function and helps in the development of effective methods of ankle joint support and rehabilitation. The exoskeleton mimics these movements to restore normal gait and improve the functionality of the muscles and ligaments surrounding the ankle joint.

Ankle biomechanics analyzes movements in three main planes: dorsal flexion (range 0° to 30°) and plantar flexion (range 0° to 50°), inversion and eversion (from ±12°), and adduction and retraction (from ±10°) [27]. These movements occur in the frontal, sagittal, and transverse planes, helping to maintain joint mobility and proper function during rehabilitation. The basic ankle joint movements, such as dorsal flexion and plantar flexion, were key parameters in the development of the new ankle rehabilitation device in this study.

### 2.2. Kinematics and Performance of Exoskeleton System

**Figure 2:**
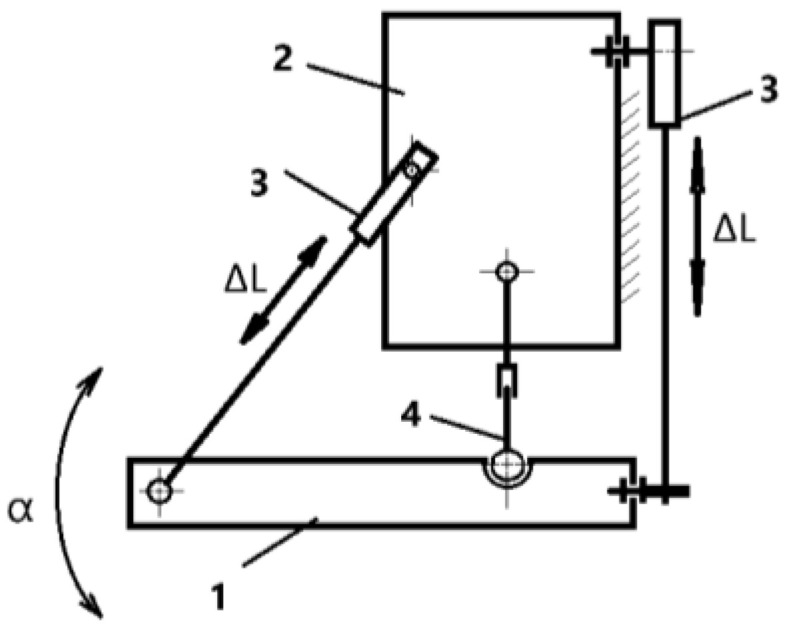
A kinematic scheme of an ankle rehabilitation exoskeleton: 1—foot platform; 2—shank platform; 3—motor; 4—spherical joint.

Important aspects of exoskeleton kinematics include key movements such as dorsiflexion and plantar flexion, which are essential for ankle rehabilitation. The exoskeleton also allows for circular movements, expanding the range of rehabilitation exercises and helping to restore the natural mobility of the joint. A notable feature is the adaptability of the device to different levels of load, allowing it to be used effectively by patients with different levels of fitness and at different stages of rehabilitation. This research employs a mixed method of qualitative and quantitative analysis to obtain accurate data. The mathematical model is based on the kinematic scheme discussed in previous studies. Results from these studies confirmed its effectiveness but revealed the need for further improvement and development of the exoskeleton to achieve greater accuracy and adaptability. The kinematic diagram of the rehabilitation device is shown in Figure 2. All necessary mathematical data were calculated according to the kinematic scheme [28,29]. The degrees of freedom (DOF) for the planar exoskeleton mechanism are determined based on the mobility equation the Chebyshev for planar systems. As stated in Equation (1), the DOF is calculated as follows:

(1) $$W=3\times \left(\;n-1\right)-2\times p_{5}-p_{4}$$

where

*n*—number of links;
*p*_5_—number of fifth-class couples (single-movement couples);
*p*_4_—number of pairs of the fourth class (two movable pairs).

For the given exoskeleton mechanism (Figure 2), the following values are used:

n=4 (foot platform, shank platform, actuator, and fixed ground);
$p_{5}=5\;$(two prismatic actuators);
$p_{4}=1$ (one spherical joint).

Thus, the DOF calculation is as follows:

W=3×(4−1)−2×(2)−1=3

According to the calculation, three degrees of freedom perform movements dorsiflexion/plantar flexion (*α*), inversion/eversion, and axial rotation. The following parameters were determined for the kinematic scheme of the rehabilitation exoskeleton in Figure 2 of the ankle joint:

The movements of the platform (1) relative to the tail of the exoskeleton (2) are characterized by a position equation, taking into account the angles of displacement of the actuator Δ*L* and rotation of the platform *α* for the legs.

(2) ΔL=f(α)

The velocity equation for this system is defined as follows:

(3) ΔL˙=J(α)×α˙

where J(α) represents the Jacobian matrix, which relates actuator displacement to ankle joint motion.

The torque equation (dynamic model) is as follows:

(4) τ=JT(α)×F

where τ is the actuator torque, and F represents the external forces acting on the exoskeleton.

Determining these parameters allows the exoskeleton to repeat the movements of the human ankle in rehabilitation processes. It can control the movement of the exoskeleton in the 3DOF.

Thus, all these calculations are required as input data for the virtual 3D model of the proposed rehabilitation device.

Figure 2 is a kinematic diagram of the exoskeleton for ankle rehabilitation. The foot platform (1) is used to place the user’s foot and allows movement of the joint. The tibia platform (2) fixes the tibia and is connected to other elements of the device. The actuator (3) is responsible for controlling the movement of the device, varying the length of the actuator (Δ*L*) to create the desired angle of motion (*α*). The articulated joint (4) provides mobility between the foot platform and the tibia, allowing smooth rehabilitation movements.

This kinematic scheme allows for the calculation of the necessary parameters for building a 3D model and further mathematical modeling of the device, including the definition of degrees of freedom.

**Figure 3:**
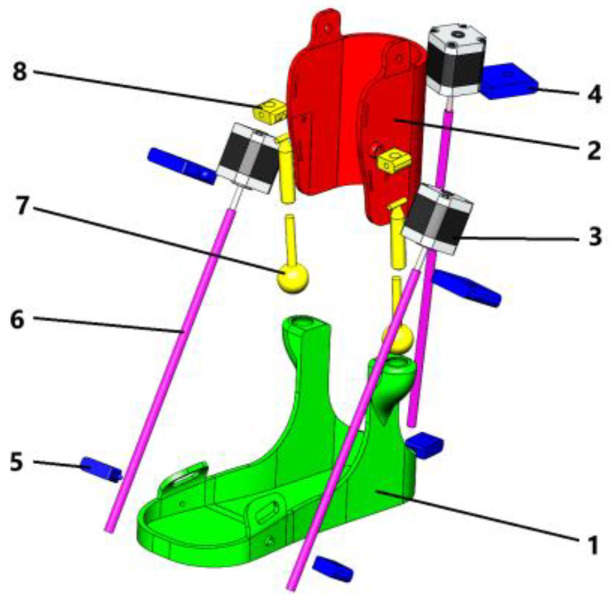
Exploded 3D model of rehabilitation exoskeleton: 1—foot platform; 2—shank platform; 3—motor; 4—motor holder; 5—screw nut; 6—screw shaft; 7—ball joint; 8—cylindrical joint.

Figure 3 shows an exploded 3D model of a rehabilitation exoskeleton designed to support and restore ankle joint movements during rehabilitation. Table 1 summarizes the characteristics of its main components.

The actuators used in ankle exoskeletons come in a variety of forms, each with distinctive properties and advantages that make them more or less suitable for specific ankle exoskeleton applications. The choice of actuator type depends on factors such as the desired range of motion, force requirements, weight limitations, and overall exoskeleton design goals [30]. Considering parameters such as power, bandwidth, strain, stress, linearity, and energy efficiency, a helical actuator was selected.

The screw actuators provide smooth and controlled movement, ensuring the exoskeleton is easy for patients to use. It also prevents sudden jerks, reducing the risk of re-injury and promoting effective recovery. Due to the self-locking effect of the screw actuators, the exoskeleton can hold the limb in position without continuous use of the motor, facilitating rehabilitation and reducing stress on the actuators. Compared with other varieties of actuators, it has high precision and stability of the mechanism. It can withstand significant loads with low power consumption.

### 2.3. Development of Exoskeleton Control System

**Figure 4:**
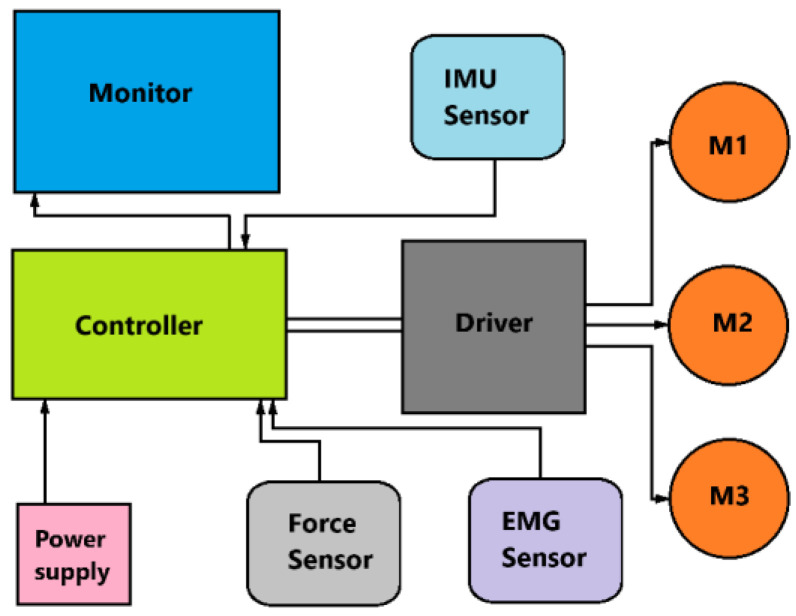
A conceptual design diagram.

Figure 4 is a control schematic for a rehabilitation exoskeleton. This control system for the rehabilitation exoskeleton consists of several key components that interact to ensure the proper operation of the device.

The main control unit is the *controller*, which processes the input signals from the sensors and determines which commands to send to the motor drives. The *driver* controls the motors based on the commands from the controller. It distributes power and control signals to the motors. The *monitor* displays information about the system operation and helps the operator monitor the parameters in real time. The *IMU Sensor* is an inertial sensor that monitors tilt angles and acceleration. It is used to measure the movement of the exoskeleton. The *force sensor* measures the load or force applied to the exoskeleton and sends the data to the controller to adjust the movement. The *EMG sensor* measures the electrical activity of the user’s muscles, allowing the system to adapt motion support based on muscle activity. The *power supply* provides electrical power to all components of the system. *Motors (M1, M2, M3)* are the actuators that perform the physical movements in the exoskeleton, controlled through the driver and controller.

**Figure 5:**
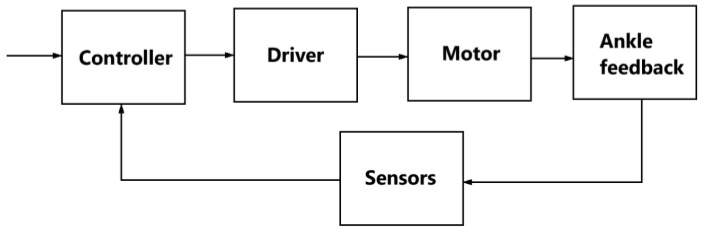
A scheme for the control design for the proposed exoskeleton.

The block diagram in Figure 5 describes the operation of the motion control system in an exoskeleton with ankle feedback. Data about the position or angle of the ankle joint is fed back to the system. This feedback allows the system to correct movement in real time, providing more accurate and adaptive motion control.

The system dynamically adapts the support of the user’s movements depending on sensor data. The EMG sensors in this case measure the electrical activation of the user’s muscles. The exoskeleton control system applies threshold values of electrical muscle activity defined for the user during calibration. When the muscle activity exceeds the defined threshold, the system interprets this as a command to start a movement. Data on the current position and orientation of the exoskeleton segments is provided by inertial measurement units (IMUs). To determine these data, mathematical models based on the kinematics and dynamics of the exoskeleton were applied to calculate the required angles and velocities of movement based on the data from the IMUs. These data are needed to adjust the exoskeleton’s movements to ensure smooth and accurate execution of specified movement trajectories during rehabilitation. Force sensors measure the force applied to the exoskeleton. IMU, force, and EMG sensors collect information about movements and loads. The data collected from the IMU, force, and EMG sensors is fed to the controller, which processes the information and determines how the exoskeleton should move. The controller sends commands to the driver, which controls the operation of the motors (M1, M2, M3). The motors create motion based on the commands, which ensures that the exoskeleton moves. During the movement, sensors collect information about the ankle condition and other motion parameters. These data are sent back to the controller, providing feedback that allows the controller to adjust motor operation and tailor the exoskeleton’s movement to the user’s needs. A monitor displays current system performance parameters for operator control.

## 3. Results

The design process for an ankle rehabilitation exoskeleton combines traditional engineering design methods with modern modeling and analysis technologies. This approach enables the creation of a more efficient and comfortable device that better aligns with the biomechanical characteristics of the ankle joint. To achieve this goal, the study utilized powerful numerical analysis and modeling tools such as SolidWorks Simulation, along with a specialized Motion Simulation module to enhance motion analysis capabilities. These tools allow for the analysis of dynamic loads acting on the rehabilitation device.

A key stage in the development of an ankle rehabilitation exoskeleton is achieving dynamic articulation (motion creation) in simulation. This enables the reproduction of natural joint movements and provides insights into load distribution and kinematic characteristics of the movements.

**Figure 6:**
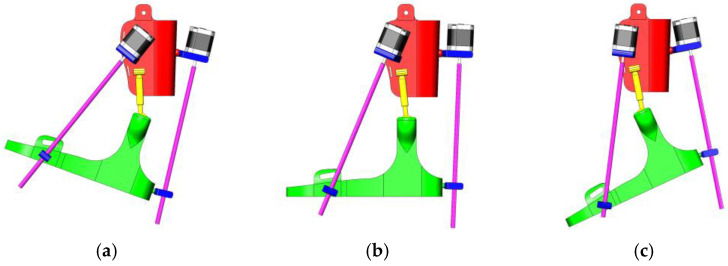
Exoskeleton positions for ankle rehabilitation: dorsal flexion (**a**), neutral position (**b**), and plantar flexion (**c**).

Figure 6 shows the three key positions of the exoskeleton for ankle rehabilitation:

(a) Dorsiflexion;
(b) Neutral position;
(c) Plantar flexion.

(a) Dorsiflexion Phase: In the dorsiflexion state, the foot is lifted upwards by an angle of 15°, which is a crucial part of natural foot movement. This movement actively engages the tibialis anterior muscles, stimulating foot elevation and expanding the patient’s range of motion. Additionally, this motion is aimed at restoring muscle activity and improving the motor functions of the foot.
(b) Neutral Position: The neutral position represents the natural resting state of the foot, where the ankle joint is positioned at a 90° angle. This position ensures stability and serves as the primary support phase during rehabilitation. Throughout the rehabilitation process, this position helps the joint and muscles adapt to normal conditions, while the exoskeleton assists in activating the patient’s leg muscles.
(c) Plantar Flexion Phase: In the plantar flexion state, the foot is lowered by 20°, mimicking the propulsion phase of walking. This movement is designed to activate the posterior leg muscles and enhance their flexibility. During rehabilitation, such movements help strengthen the patient’s leg muscles, increase movement amplitude, and improve limb coordination.

**Figure 7:**
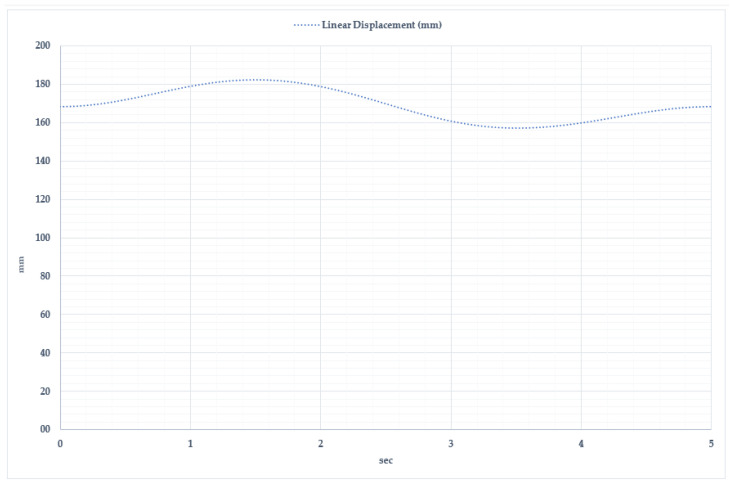
Time dependency of linear displacement during dorsiflexion and plantar flexion movements.

Figure 7 presents the linear displacement parameters of dorsiflexion and plantar flexion movements analyzed over time. The start of measurement or the so-called reference point for this movement is the neutral position of the foot, shown in Figure 6b. This position shows the natural state of the foot when the ankle joint angle is 90°. All linear displacement measurements are made along the vertical axis (Y) relative to this point. Experimental and modeling studies determined that the linear displacement of the exoskeleton’s moving elements varies between 155 mm and 185 mm. As observed in the graph, the total movement cycle lasts approximately 5 s and consists of two main phases.

Dorsiflexion Phase: At the beginning of the movement, the exoskeleton system lifts the foot upwards. The maximum displacement reaches 182–185 mm within 1.5–2 s. During this phase, the actuators and motors actively ensure the required movement amplitude.

Plantar Flexion Phase: In this phase, the foot moves downward, reaching its lowest displacement (155–160 mm) within 3.5 s. This movement simulates the foot’s push-off phase, while the motion system adapts to dynamic load variations by adjusting torque.

**Figure 8:**
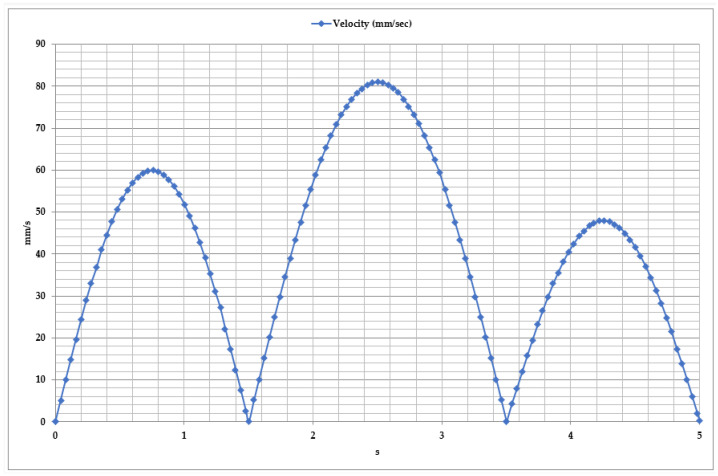
Time dependency of velocity during dorsiflexion and plantar flexion movements.

To ensure the efficient operation of the exoskeleton, it is crucial to analyze the kinematic parameters of movement, including the time-dependent variation in velocity, as shown in Figure 8. This figure focuses on determining the velocity profile of dorsiflexion and plantar flexion movements. The velocity analysis revealed that a complete movement cycle lasts approximately 2 s. From the graph, three key phases of motion can be identified.

Dorsiflexion Phase: As the foot moves upward, the velocity increases to 60–70 mm/s before gradually decreasing to zero at the end of the movement. This indicates the capability of the exoskeleton’s mechanical actuators and motors to control movement accurately.

Plantar Flexion Phase: As the foot moves downward, the velocity reaches approximately 90 mm/s, which is slightly higher than in the dorsiflexion phase. This movement simulates the foot’s propulsion phase, with the exoskeleton using a dynamic adaptation algorithm to manage this process effectively.

Once the first movement cycle is completed, a new cycle begins. However, in the third cycle, a decrease in velocity (approximately 50 mm/s) is observed, which may indicate the influence of external factors such as load variations and control algorithm adjustments on the system’s dynamics.

**Figure 9:**
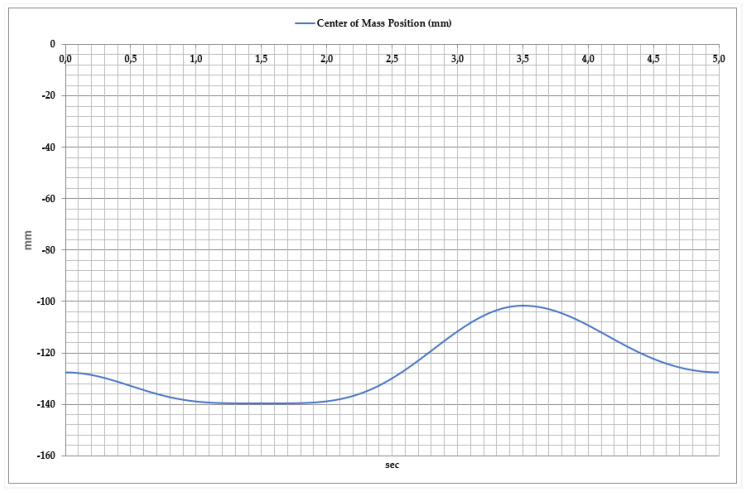
Time dependency of center of mass position during dorsiflexion and plantar flexion movements.

To ensure the stability and efficient operation of the exoskeleton, it is essential to analyze the center of mass (CoM) position dynamics during movement, as illustrated in Figure 9. This figure directly influences the movement coordination of the exoskeleton and the effectiveness of rehabilitation exercises. Analysis of the center of mass displacement revealed that during the exoskeleton’s movement, the CoM position varies between −160 mm and −100 mm. This movement can be divided into two main phases.

Dorsiflexion Phase: As the foot moves upward, the center of mass gradually rises. The maximum CoM position reaches approximately −100 mm, peaking at around 3.5 s. During this phase, the exoskeleton’s mechanical system dynamically adapts to facilitate foot elevation.

Plantar Flexion Phase: As the foot moves downward, the center of mass shifts downward again, returning to its initial position of approximately −120 mm. This movement represents the foot’s downward push and the redistribution of load during motion.

**Figure 10:**
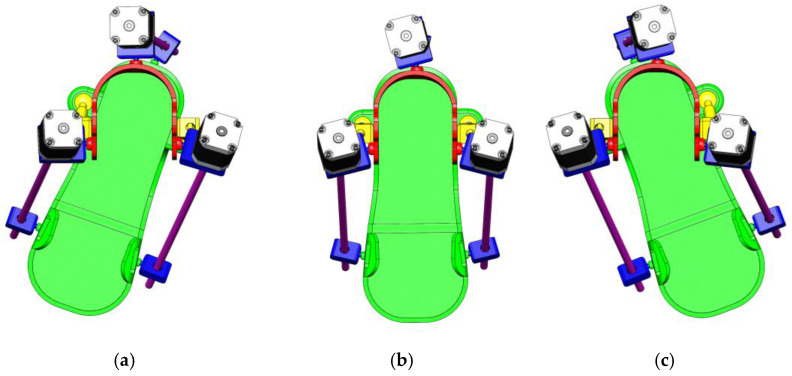
Position of exoskeleton in different phases of lateral movement of foot: (**a**) inward adduction position; (**b**) neutral position; (**c**) outward adduction position.

Figure 10 illustrates the exoskeleton’s ability to support lateral movements of the ankle joint. These movements include inversion and eversion, where the foot tilts inward and outward, respectively. This functionality is crucial in comprehensive rehabilitation, as it contributes to restoring foot stability, movement amplitude, and full motor function.

(a) Inward Adduction Position—Inversion: In this position, the foot tilts inward, raising the medial (inner) side of the sole. This movement is used to strengthen the internal ligaments and muscles of the foot, improving joint stability.
(b) Neutral Position—Resting State: In this configuration, the ankle joint remains in its natural balanced state, with the foot fully relaxed. This position serves as the initial and intermediate reference point during rehabilitation exercises.
(c) Outward Adduction Position—Eversion: In this case, the foot tilts outward, raising the lateral (outer) side of the sole. This movement helps activate the external foot muscles and ensures a full range of motion, essential for restoring ankle mobility.

**Figure 11:**
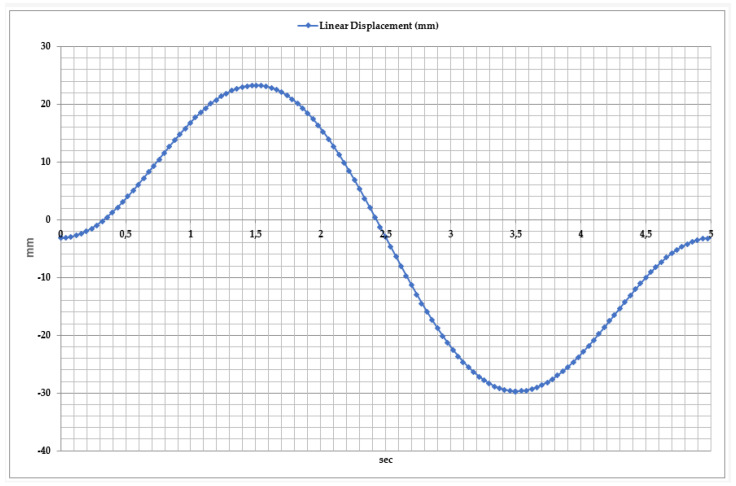
Time dependency of linear displacement during inversion and eversion movements.

To provide comprehensive rehabilitation of the ankle joint, the dynamics of inversion and eversion movements are analyzed in Figure 11. It characterizes the lateral movement of the foot, where the linear movement of the exoskeleton is measured along the horizontal axis (X). The measurement reference point in this case is the neutral position of the foot when it is positioned without inward or outward tilt, maintaining a resting state. An inward tilt of the foot corresponds to an inversion, according to the range of positive values in the graph. Negative values correspond to an outward tilt of the foot, eversion.

Studies have shown that during inversion and eversion movements, the linear displacement of the exoskeleton varies from −35 mm to +25 mm. The graph identifies three key phases of movement.

Inversion Phase: In the first 2 s, the foot tilts inward, reaching a maximum displacement of 25 mm. This movement activates the medial foot muscles and improves lateral stability.

Neutral Position: Between 2 and 3 s, the foot returns to its initial position, balancing the two movement phases. During this phase, the exoskeleton actuators regulate dynamic loads, ensuring smooth movement execution.

Eversion Phase: From 3 to 4.5 s, the foot tilts outward, reaching a displacement of −35 mm. This movement is necessary to activate the lateral foot muscles and ensure a full range of motion.

**Figure 12:**
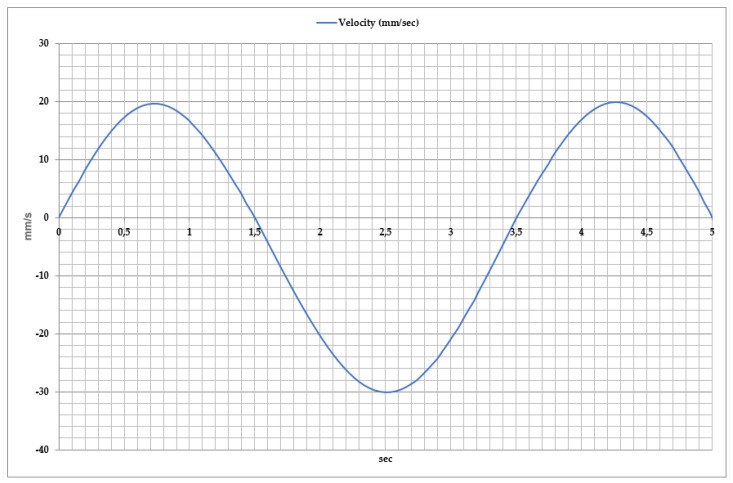
Time-dependent velocity during inversion and eversion movements along the vertical axis (Y).

To ensure comprehensive rehabilitation of the ankle joint, the velocity variations in inversion and eversion movements are analyzed in Figure 12. This parameter allows for the assessment of the exoskeleton’s movement efficiency and provides insights into the patient’s functional recovery process. Studies have shown that during inversion and eversion movements, velocity ranges from −35 mm/s to +25 mm/s. The movement dynamics consist of three key phases.

Inversion Phase: In the first second, velocity reaches positive values (approximately 20 mm/s) as the foot tilts inward. During this phase, the exoskeleton’s control system ensures proper foot elevation, while muscle activity increases.

Eversion Phase: Between 1.5 and 3 s, velocity shifts to negative values, reaching −35 mm/s, indicating the outward tilting of the foot. This phase helps enhance lateral stability and activate the lateral foot muscles.

Cycle Repetition: Between 3 and 5 s, velocity returns to positive values, initiating a new inversion phase. This process ensures the stability of movement dynamics.

**Figure 13:**
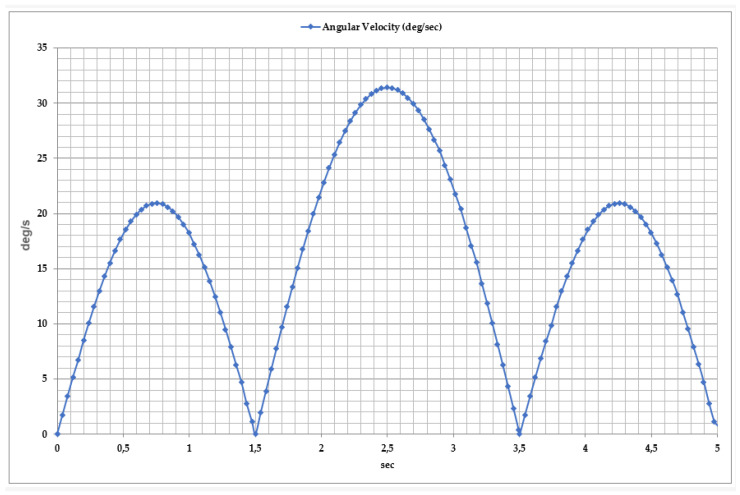
Time dependency of angular velocity during inversion and eversion movements.

The parameter shown in Figure 13 allows for the assessment of the exoskeleton’s ability to control movement and provides insights into the patient’s functional recovery process. Studies have demonstrated that the angular velocity varies from 0°/s to 30°/s. The movement dynamics consist of three key phases.

Inversion Phase: During the first second, the angular velocity reaches 20°/s, and the foot tilts inward. At this stage, the exoskeleton’s control system ensures proper foot elevation, while muscle activity increases.

Eversion Phase: Between 1 and 2 s, the velocity decreases to zero, initiating the eversion movement. This phase helps improve lateral stability and activate the lateral foot muscles.

Movement Cycle Repetition: From 2 to 5 s, the velocity increases again, initiating new inversion and eversion phases. The consistent and controlled variation in angular velocity enhances movement efficiency.

**Figure 14:**
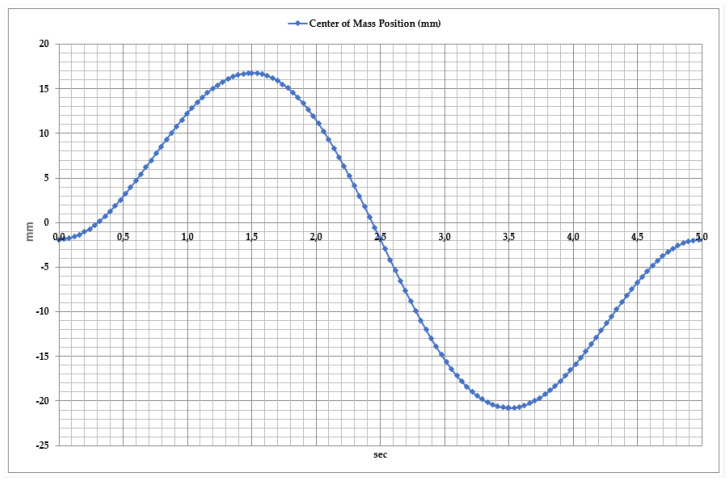
Time-dependent velocity during inversion and eversion movements along the axis (Z).

Figure 14 illustrates that during inversion and eversion movements, the velocity varies from −35 mm/s to +25 mm/s. The movement dynamics consist of three key phases.

Inversion Phase: During the first second, the velocity reaches positive values (approximately 20 mm/s) as the foot tilts inward. At this stage, the exoskeleton’s control system ensures proper foot elevation, while muscle activity increases.

Eversion Phase: Between 1.5 and 3 s, the velocity shifts to negative values, reaching −35 mm/s, indicating the outward tilting of the foot. This phase helps enhance lateral stability and activate the lateral foot muscles.

Cycle Repetition: Between 3 and 5 s, the velocity returns to positive values, initiating a new inversion phase. This process ensures the stability of movement dynamics.

**Figure 15:**
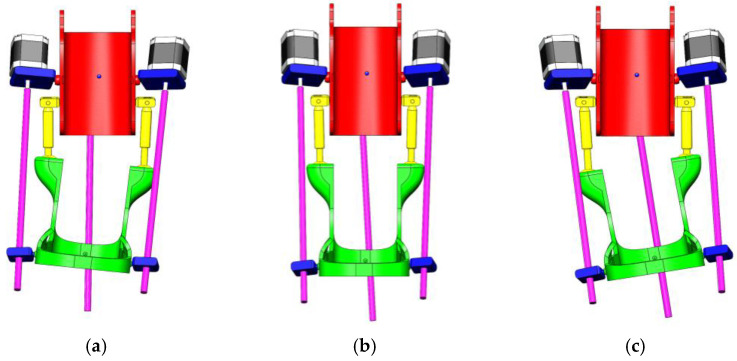
Ankle exoskeleton positions: valgus (**a**), neutral (**b**), and varus (**c**).

Figure 15 illustrates the exoskeleton’s ability to support abduction (valgus) and abduction (varus) movements. These movements are responsible for the medial and lateral motion of the foot and play a crucial role in restoring lateral stability of the ankle joint.

(a) Valgus (Abduction) Position: In this position, the foot tilts outward within a range of 5–15°, deviating laterally relative to the Y-axis. This movement stretches the inner ligaments of the foot and helps improve lateral stability.
(b) Neutral Position: In this configuration, the ankle joint remains in a naturally balanced state, with the foot in a neutral position. This serves as the starting and intermediate reference point during rehabilitation.
(c) Varus (Adduction) Position: Here, the foot tilts inward within a range of 5–15°, deviating medially relative to the Y-axis. This movement restores medial stability and ensures a full range of motion for the foot’s lateral movements.

**Figure 16:**
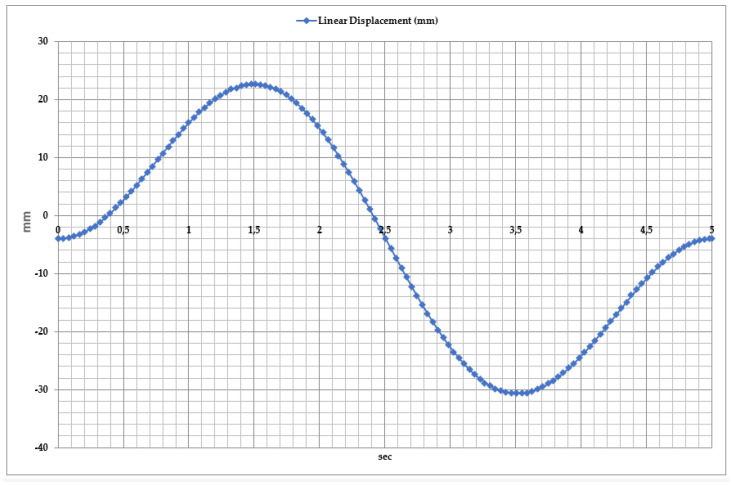
Time dependency of linear displacement during abduction and adduction movements.

To ensure comprehensive rehabilitation of the ankle joint, the linear displacement dynamics of abduction (valgus) and adduction (varus) movements were analyzed, as shown in Figure 16. These movements are responsible for lateral foot motion and contribute to restoring joint stability.

Studies have demonstrated that the linear displacement of the exoskeleton varies from −35 mm to +25 mm. The movement consists of three key phases.

Abduction Phase: During the first 2 s, the foot shifts outward by 25 mm, indicating lateral movement of the foot. At this stage, the exoskeleton’s actuators help maintain lateral stability and control the outward movement of the foot.

Neutral Position: Between 2 and 3 s, the foot returns to its initial position, balancing the two movement phases. This phase allows for an evaluation of the exoskeleton’s ability to regulate dynamic loads.

Adduction Phase: From 3 to 4.5 s, the foot moves inward by 35 mm, indicating medial movement of the foot. In this phase, the exoskeleton control system ensures the proper execution of inward foot movement.

The exoskeleton’s ability to effectively control linear displacement allows for rehabilitation exercises to be adjusted according to the patient’s physiological capabilities. Ensuring smooth and controlled execution of movements contributes to strengthening foot muscles, improving joint flexibility, and restoring the patient’s range of motion.

**Figure 17:**
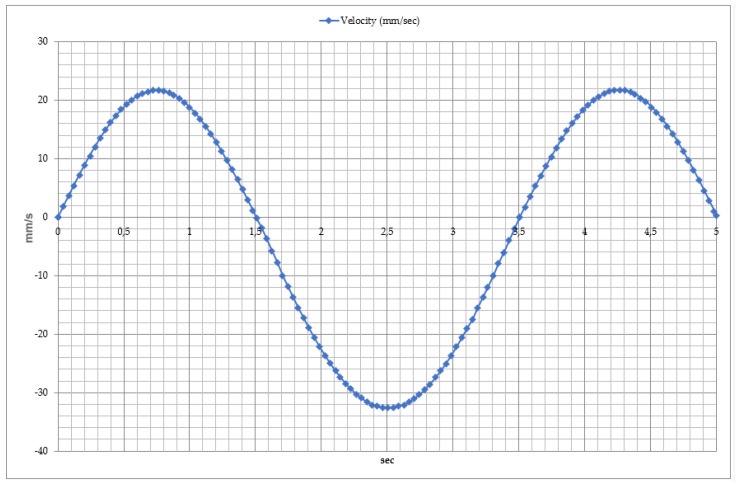
Time dependency of velocity during abduction and adduction movements.

To ensure comprehensive rehabilitation of the ankle joint, the velocity dynamics of abduction (valgus) and abduction (varus) movements were analyzed, as shown in Figure 17. This parameter allows for the assessment of the exoskeleton’s ability to control movement and provides insights into the patient’s functional recovery process.

Studies have demonstrated that velocity varies from −35 mm/s to +25 mm/s. The movement dynamics consist of three key phases.

Abduction Phase: During the first second, velocity reaches 20 mm/s, and the foot moves outward. At this stage, the exoskeleton’s control system regulates outward foot movement, playing a crucial role in the rehabilitation process.

Adduction Phase: Between 1.5 and 3 s, velocity shifts to negative values, reaching −35 mm/s, indicating the inward movement of the foot. This phase helps enhance lateral stability and activate the medial foot muscles.

Cycle Repetition: From 3 to 5 s, velocity returns to positive values, initiating a new abduction phase. The consistent and controlled variation in velocity improves movement efficiency.

**Figure 18:**
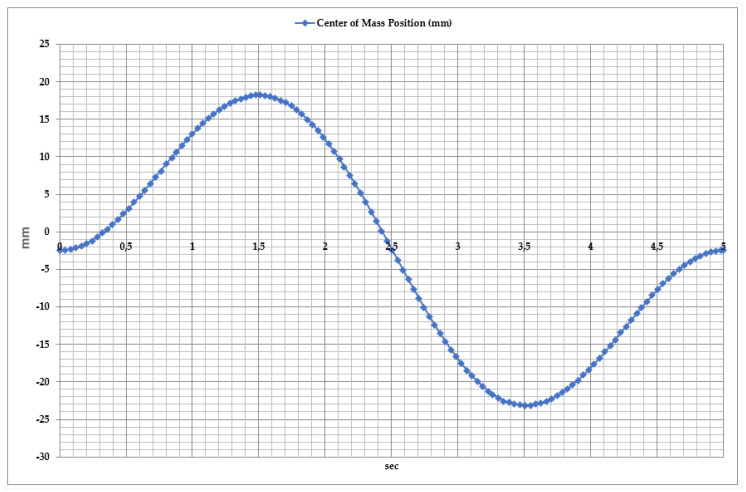
Time dependency of center of mass variation during abduction and adduction movements.

To ensure the stability and efficient operation of the ankle exoskeleton, the center of mass (CoM) variations during abduction (valgus) and adduction (varus) movements were analyzed, as shown in Figure 18. This parameter is used to evaluate movement coordination, optimize the exoskeleton’s control algorithms, and assess the patient’s rehabilitation progress. Studies have shown that the center of mass displacement varies from −30 mm to +25 mm. The movement dynamics can be divided into three key phases.

Abduction Phase: During the first 2 s, the center of mass shifts up to +25 mm, indicating the outward tilt of the foot. This phase is aimed at restoring lateral foot movements and enhancing the exoskeleton’s ability to control motion.

Adduction Phase: Between 2 and 3.5 s, the center of mass decreases to −30 mm, indicating the inward tilt of the foot. At this stage, the exoskeleton’s dynamic adaptation capability and balance control efficiency are evaluated.

Cycle Repetition: Between 4 and 5 s, the center of mass returns to its initial position, ensuring movement stability and proper execution.

Experimental data obtained during the modeling and simulation of the exoskeleton’s operation show that the device provides smooth and controlled movements, which is critical for the rehabilitation of patients with varying degrees of functional impairment. Smooth changes in motor torque and the cyclic nature of the angular movements of the exoskeleton components confirm that the system can adapt to the individual needs of patients, thereby providing optimal recovery conditions. The results for translational velocity and angular displacement demonstrated stability and repeatability throughout testing. This suggests that the exoskeleton not only replicates natural movements but does so with a high degree of accuracy and control, which is essential for the successful recovery of the rehabilitated patient.

## 4. Discussion

**Figure 19:**
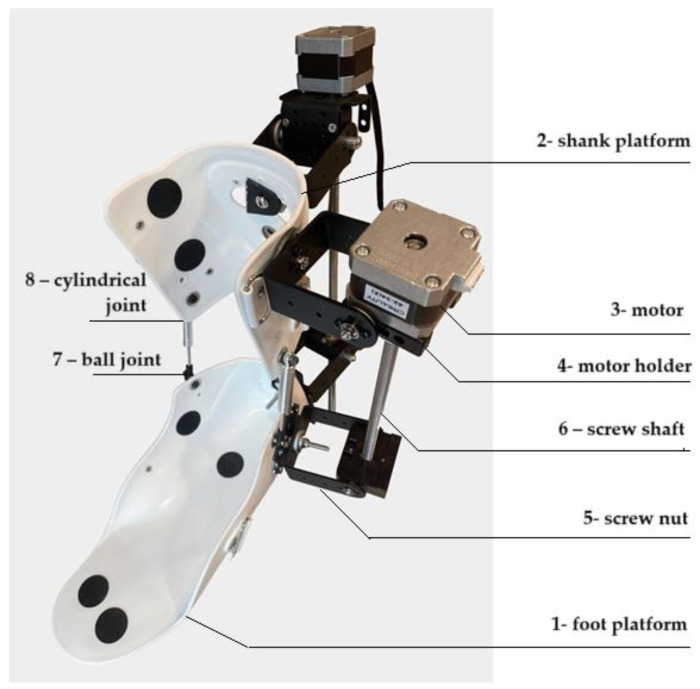
Preliminary prototype of proposed rehabilitation device (frontal view).

The presented novel ankle exoskeleton design is shown in Figure 19. This screw shaft-based design is expected to have a high degree of precision and control. This increases the efficiency of its application in ankle rehabilitation processes. Advanced 3D printing technology was applied in the assembly process of the exoskeleton design. Lightweight, flexible, biodegradable PLA material was used to form the main body.

The assembly process of the prototype ankle exoskeleton module consists of successive connections of mechanical and electronic components. This ensures functionality and reliability in use. First, the base of the structure must be assembled. The foot platform (1) is fixed to provide stable support for the foot. To connect the lower part to the upper components of the exoskeleton, the shank platform (2) is attached to the platform. At this stage, to ensure safety, it is necessary to make sure that these elements are installed symmetrically and securely fixed. Next, the mechanical part is mounted. For the transition of rotary motion into translational motion, the screw shaft (6) is fixed with the screw nut (5) attached to it. At this stage it is necessary to ensure that the exoskeleton shaft rotates without jamming. Afterwards, the motors (3) are fixed into the structure on special holders (4). The motors must be properly aligned with the propeller shaft for efficient motion transmission. To ensure flexibility and mobility of the exoskeleton, a ball joint (7) is installed between the screw mechanism and the platform shank. To ensure stability and correct movement, a cylindrical joint (8) is fixed. Electronic components, Arduino Mega (Arduino Mega 2560, Funduino, China), drivers TB6600, and sensor ACS 712 are located in a special housing of the control unit. The motors are connected to the TB6600 driver to connect a power supply to the system and to provide control. The ACS 712 sensor in this case monitors the current in the system. All elements are connected to the Arduino Mega, which performs the control functions. It is important to check the correct connection of all wires and electronic elements. At the final stage, calibration and functional testing of the device are carried out. In further studies, it is planned to check the operation of motors and mechanical connections. Also, the smoothness and accuracy of the movements of the platform are checked. Then, the software is configured on Arduino Mega to control the modes of exoskeleton operation. After customization, the device is tested with different operating parameters to confirm its functionality and compliance with biomechanical requirements. This will ensure reliable operation and durability of the device. The characterization of the main key components of the exoskeleton prototype is summarized in Table 2.

**Figure 20:**
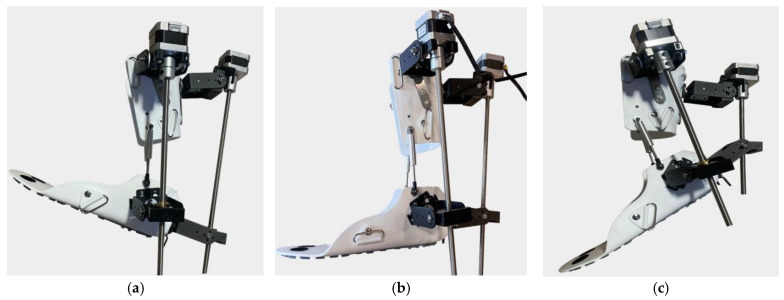
The prototype ankle exoskeleton positions for ankle rehabilitation: dorsal flexion (**a**), neutral position (**b**), and plantar flexion (**c**).

The main achievement of this research is the biomechanical compatibility of the exoskeleton prototype with the human ankle joint. The proposed compact design makes it possible to use it in clinical and home rehabilitation settings due to its ease of use. Experimentally, without considering user parameters, the exoskeleton prototype effectively performed dorsal, neutral, and plantar flexion movements. The results of the performed physical tests of the exoskeleton prototype confirmed the consistency with the obtained results of the virtual tests. Figure 20 shows the basic positions of movements performed by the exoskeleton prototype necessary for ankle joint rehabilitation.

In this case, the motor rotation speed control is carried out through the Arduino Mega controller. To raise the lower part of the exoskeleton prototype platform in the process of dorsiflexion, the controller increases the rotation speed. Accordingly, to ensure the smooth release of the lower part of the exoskeleton prototype platform, this speed will be decreased. In the case of deviation from the set movement trajectory, the controller will correct the motor rotation speed to achieve the correct trajectory.

**Figure 21:**
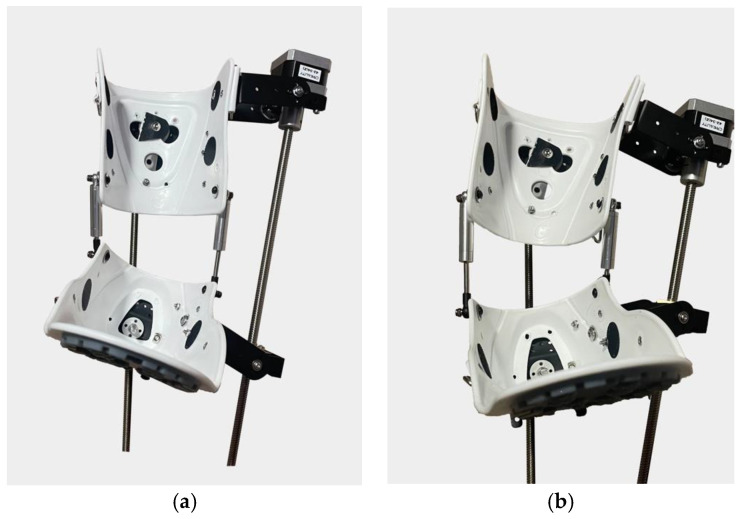
The prototype ankle exoskeleton positions: valgus (**a**) and varus (**b**).

Lateral stability also plays an important role in the ankle rehabilitation process. In this phase of the study, the ability of the exoskeleton prototype to support lateral movements of the valgus (a) and varus (b) foot was evaluated. Figure 21 shows the two key lateral movement positions of the exoskeleton prototype. In the valgus position (a), the lower platform of the exoskeleton is deflected outwards between 5° and 15° relative to the Y-axis. In the varus position (b), the lower platform of the exoskeleton tilts inward (medially) in a range of 5° to 15° relative to the Y-axis. The results obtained from physical testing of the exoskeleton prototype confirmed biomechanical compatibility in performing lateral movements. This makes it a promising tool for rehabilitation in clinical practice.

During the tests, the device demonstrated the ability to reproduce natural movement amplitudes within a specified range. In the rehabilitation process, this helps to accelerate the recovery of ankle joint mobility. The smoothness of the range of motion also reduces the risk of re-injury and prevents overloading of other joints.

Despite the encouraging results, the current design has some limitations. Virtual testing of the conceptual model and physical testing of the exoskeleton prototype were conducted without taking into account the individual parameters of the user. In this case, the real rehabilitation conditions may have a discrepancy with the obtained study results. In future research, it is planned to optimize the design to reduce weight and increase user comfort. Also, further research will be devoted to conducting clinical trials to evaluate the performance of the device in real conditions.

## 5. Conclusions

In this research, an exoskeleton model for ankle rehabilitation was developed and tested. The modeling and simulation results showed high efficiency and precise and smooth motion control. The use of a screw actuator allows the joint to be held in a given position without constantly switching on the motor, which increases the efficiency of the device.

Mathematical modeling and virtual testing demonstrated the high accuracy of the motion control of the system. The smoothness of the force torque and angular displacement changes confirmed the high control accuracy, which is especially important when working with patients requiring gradual motor recovery. After virtual testing, the assembly of the prototype ankle exoskeleton was realized, which consists of sequential connections of mechanical and electronic components. The prototype assembly process included assembly of the design basis, assembly of the mechanical part, and connection of electronic components of the control unit: Arduino Mega, TB6600 driver, and ACS 712 sensor. To ensure safety, the mechanical part was checked to ensure that all wires and electronic components were connected correctly. Standard modules such as Arduino Mega for control, TB6600 as a motor driver, and ACS 712 sensor for current control are used to build the control unit. These materials and components provided a combination of functionality, lightness, and cost-effective design, making the exoskeleton suitable for testing and further use in rehabilitation. This shows great potential for clinical application due to its compactness, low cost, and ability to adapt to individual patient needs. After all stages of testing, the device can be used both in specialized rehabilitation centers and at home to support and accelerate the recovery process. The study highlights the importance of using robotic exoskeletons in the rehabilitation of patients with musculoskeletal injuries and diseases and demonstrates the potential for future advances in rehabilitation technology.

Further efforts of the authors will focus on improving the proposed design and on conducting experimental studies to determine the main characteristics and parameters. Tests on the functionality of the device will be carried out, including performance evaluation under real operating conditions. The use of computer modeling with practical verification through prototyping offers great potential for the development of advanced technologies in the field of rehabilitation and assistive devices.

## Figures and Tables

**Table 1:** Main characteristics of components 3D model of rehabilitation exoskeleton.

№	Title	Designation
1	foot platform	A support part of the exoskeleton that provides stability during movements.
2	shank platform	Fixes the user’s lower leg and connects it to other elements of the device, maintaining the correct position of the leg.
3	motor	The main drive element for controlling exoskeleton movements.
4	motor holder	Attaches the motor to the structure, ensuring its stable position and transmission of force to other parts of the exoskeleton.
5	screw nut	Works in tandem with the propeller shaft to convert rotary motion into linear motion.
6	screw shaft	Converts the rotary motion of the motor into linear motion for precise control in rehabilitation devices. The helical gearing provides smooth and controlled movements.
7	ball joint	Provides freedom of movement in several planes, allowing natural joint movements to be reproduced.
8	cylindrical joint	Enables the rotational movements that are necessary for the correct functioning of the mechanism.

**Table 2:** Parameters of main prototype units.

Component	Commercial Name	Voltage	Mass	Max Force/Torque	Speed
Arduino board	Mega 2560 23	7–12 V	37 g	—	—
Actuator	Mini Electric	12 V	150 g	100 N	20 mm/s
Servomotor	NEMA 17	4.8–7.2 V	280 g	150 N–cm	461.5 deg/s
Driver	TB6600	4.75–12 B	125 g	—	—
IMU	BMI16025	3–5 V	2 g	—	—
EMG	Grove—EMG Detector	3.3–5 B	45 g	—	—

## Data Availability

The data presented in this study are available on request from the corresponding author.
